# Access to Cancer Screening in People with Learning Disabilities in the UK: Cohort Study in the Health Improvement Network, a Primary Care Research Database

**DOI:** 10.1371/journal.pone.0043841

**Published:** 2012-08-29

**Authors:** David P. J. Osborn, Laura Horsfall, Angela Hassiotis, Irene Petersen, Kate Walters, Irwin Nazareth

**Affiliations:** 1 Mental Health Sciences Unit, University College London, London, United Kingdom; 2 Research Department of Primary Care and Population Health, University College London, London, United Kingdom; University of Pennsylvania, United States of America

## Abstract

**Objectives:**

To assess whether people with learning disability in the UK have poorer access to cancer screening.

**Design:**

Four cohort studies comparing people with and without learning disability, within the recommended age ranges for cancer screening in the UK. We used Poisson regression to determine relative incidence rates of cancer screening.

**Setting:**

The Health Improvement Network, a UK primary care database with over 450 General practices.

**Participants:**

Individuals with a recorded diagnosis of learning disability including general diagnostic terms, specific syndromes, chromosomal abnormalities and autism in their General Practitioner computerised notes. For each type of cancer screening, a comparison cohort of up to six people without learning disability was selected for each person with a learning disability, using stratified sampling on age within GP practice.

**Main outcome measures:**

Incidence rate ratios for receiving 1) a cervical smear test, 2) a mammogram, 3) a faecal occult blood test and 4) a prostate specific antigen test.

**Results:**

Relative rates of screening for all four cancers were significantly lower for people with learning disability. The adjusted incidence rate ratios (95% confidence intervals) were Cervical smears: Number eligible with learning disability = 6,254; IRR = 0.54 (0.52–0.56). Mammograms: Number eligible with learning disability = 2,956; IRR = 0.76 (0.72–0.81); Prostate Specific Antigen: Number eligible = 3,520; IRR = 0.87 (0.80–0.96) and Faecal Occult Blood Number eligible = 6,566; 0.86 (0.78–0.94). Differences in screening rates were less pronounced in more socially deprived areas. Disparities in cervical screening rates narrowed over time, but were 45% lower in 2008/9, those for breast cancer screening appeared to widen and were 35% lower in 2009.

**Conclusion:**

Despite recent incentives, people with learning disability in the UK are significantly less likely to receive screening tests for cancer that those without learning disability. Other methods for reducing inequalities in access to cancer screening should be considered.

## Introduction

People with learning disabilities have high rates of physical morbidity and co-occurring physical disorders [1], [2]. In 2006, the UK Disability Rights Commission (DRC; now Equality and Human Rights Commission) highlighted inequalities, describing “diagnostic overshadowing” whereby professionals may be distracted from physical health conditions because of the primary diagnosis of a learning disability [3], [4]. The report called for better access to physical health care in this group of patients. Furthermore, health and social care professionals have failed to meet the medical needs of individuals with learning disabilities when admitted to hospital [5]. These health inequalities may be widening and health and social services have a role to ensure that routine physical services including cancer screening are provided equitably, are targeted to those with the greatest needs and are acceptable to those who need them most. The DRC recommended that a register of those with learning disability should be instigated in UK in primary care [4]. This register had also been recommended in the Department of Health white paper “valuing people” in 2001 [6] ^,^ with an expectation of compliance by 2004. A learning disability register has been part of the English primary care Quality Outcomes Framework (QOF), a financial incentive scheme for General Practitioners to improve quality of care, for adults over 18, since 2006/7. It is recommended that people on the register should receive an annual physical health check, but the content of the check is not specified. In 2008, the Department of Health for England made funding available for general practices to provide health checks for people with learning disabilities, but uptake by individual practices was optional, as part of a Directed Enhanced Service [7].

The UK NHS Cancer Screening Programmes offer age-targeted screening to the UK general population, based on the best available evidence regarding the effectiveness and risks of screening tests at different ages. They published guidance in 2006 to improve access to both breast and cervical screening programmes for people with learning disability [8]. This emphasised that people with learning disability should routinely be offered breast and cervical screening. In particular they stressed that no assumption should be made that these groups are not at risk of cervical cancer, on account of presumed sexual inactivity. Specific health promotion materials are available for people with learning disability, including picture books which explain both cervical and breast screening [8].

We aimed to explore whether rates of cancer screening differed in people with learning disability compared to people without such a diagnosis, in primary care. Our primary focus was the established screening programmes, namely cervical and breast cancer. Our secondary outcomes were screening rates for prostate and bowel cancer. We also aimed to explore whether any differences in cancer screening rates had changed over the last decade, and whether they varied by geographical social deprivation. We hypothesised that for the established screening programmes (cervical and mammography) rates of screening would be lower in people with learning disability. We also hypothesised that other screening rates for bowel and prostate cancer would be lower and that differences in the established screening programmes would have narrowed in more recent years and that disparities would be more marked in areas of greater social deprivation.

## Methods

### Ethics Statement

The Health Improvement Network (THIN) scheme of providing anonymised data to researchers was approved by the NHS South-East Multi-centre Research Ethics Committee in 2002. Further, this study received scientific approval from THIN scientific review committee, reference 10-016.

### Study Design

Retrospective cohort studies.

### Setting

United Kingdom primary care.

### Data Source

The Health Improvement Network (THIN), a primary care research database which extracts anonymised data entered during routine clinical practice, from general practices across the United Kingdom [9]. At the time this study was conducted the database included over 9 million patients, with more than 50 million years of patient data from over 450 general practices. In 2009, the active patients in THIN represented approximately 6% of the UK population. The distribution of THIN practices across UK countries reflects the distribution of the UK population. Therefore 75% of the practices were located in England, 5% in Northern Ireland, 13% in Scotland and 6% in Wales. In UK, health care is free and 98% of the population is registered with a general practice. Learning Disability is one of the conditions for which a disease register is incentivised by QOF, and therefore identification of known cases of learning disability in GP data is likely to be relatively complete since the introduction of this register in 2006.

THIN includes information for each individual on diagnoses, prescriptions, referrals, some risk factors for poor health including smoking, and local area deprivation (Townsend score [10]). GPs and practice staff use a Read code system to enter diagnoses, symptoms, investigations (including screening tests) and lifestyle information into the electronic clinical notes. Read codes are a well established hierarchical coding system, which provide standardised options for entering different diagnoses and other clinical information, and the codes are aligned to international medical coding systems such as the WHO ICD coding system. The Read code system became accepted as a standard in UK primary care in 1988 [11].

### Participants

For each type of cancer screening, such as cervical or breast screening, the age eligibility criteria for screening differ. Therefore we created four separate cohorts of people with learning disability who met these eligibility criteria (one for each of type of cancer), and also extracted data regarding four stratified random comparison cohorts of people without learning disability diagnoses who were also eligible for the screening. The age eligibility criteria and recommendations regarding frequency of screening also vary by country within the UK and therefore we matched our inclusion criteria to these recommendations. These inclusion criteria are specified below for each type of cancer screening.

For all participants we only included people after their general practice had met predefined data quality criteria within THIN [12] and who had a minimum of six months follow-up data, between 1999 and December 2009.

#### People with learning disability (the exposed cohorts)

We identified all people with a diagnostic record for a learning disability. We developed Read code lists for these diagnoses using methodology previously described [13]. We included general terms for learning disability (including QOF codes), as well as terms relating to 1) autism including Asperger syndrome and 2) chromosomal abnormalities such as Down syndrome and Fragile X syndrome. A full list of the terms included are available from the authors.

#### People without learning disability (the unexposed cohorts)

For each learning disability cohort, we extracted data regarding up to six comparison participants of the same sex who did not have a diagnosis of a learning disability in their practice record. Selecting a greater number of unexposed participants maximises the statistical power of a study, which is particularly helpful in a situation where the number of exposed individuals (ie with learning disability) is fixed. We use random stratified sampling within five-year age bands from the same practice to ensure these comparison cohorts were similar in terms of age and practice-level variables.

### Age Inclusion Criteria and Follow-up Period for Different Cancer Screening Programmes

There are different NHS cancer screening programmes for cervical, breast and bowel cancer in the UK, which have varied over time and in the different UK countries. There is not a cancer screening programme for prostate cancer, but men are given information in a “Prostate Specific Antigen Informed Choice Programme”. The age criteria we employed reflect these differences, as detailed below.

#### 1) Cervical cancer screening cohorts

In England, before 2003, the recommended frequency of screening in England ranged between 3 and 5 years for women aged 20 to 65 years. A new cervical cancer screening program began in 2003 for all women aged between 25 and 65. The screening frequency differed by age, being every 3 years for women between 25 to 49 years of age and every five years for those aged 50 to 64. In Scotland cervical screening is routinely offered every three years to women aged between 20 and 60 years of age. In Wales screening is routinely offered every three years to women aged between aged 20 to 64. In Northern Ireland, all women between the ages of 20 and 64 are invited every five years.

We started follow-up for women from the latest date of GP registration, their 20^th^ birthday (or 25^th^ for English practices after 2003) or January 1st 1998. Their exit date from the cervical screening cohort was defined as earliest date of leaving the general practice, their 65^th^ birthday (60th in Scotland). We also stopped follow-up when a reason for a change in cervical screening frequency was recorded in their clinical record including hysterectomy, testing positive for Human Papilloma Virus (HPV), the identification of precancerous cells, a diagnosis of cervical cancer or death.

For cervical screens, the general practice records allow an indicator for whether a patient should be excluded from screening, together with the date of the exclusion. A reason for excluding patients can also be recorded, although in practice this is completed for less than a third of patients. The reasons for exclusion can include reaching upper age limit for screening, hysterectomy, being considered “inappropriate for screening”, or patient refusal. Our main analysis omitted these excluded patients. However we did explore the extent to which GP exclusions had an effect on differences in cervical screening rates. We did this by also calculating a relative rate of screening which included all patients in the cohort, irrespective of whether the GP had excluded them from cervical screening.

Exclusion indicators are not available within the Read Code system for the other screening tests.

#### 2) Breast cancer screening cohorts

Since 1993, women in England, Ireland, Scotland and Wales aged between 50 and 64 years have been automatically invited for mammograms every three years.

In the breast screening cohort we began follow up at the latest date of registering at the general practice, reaching their 50^th^ birthday or January 1st 1998. Women left the cohort at the earliest date of the following: leaving the general practice, 64^th^ birthday, or a medical diagnosis of mastectomy, precancerous cells, breast cancer or death.

#### 3) Bowel cancer screening cohorts

The NHS Bowel Cancer Screening Programme in England was initiated in July 2006. It offers screening every two years to all men and women aged 60 to 69. People over 70 can request a screening kit. Similar programmes are being implemented in Scotland and Wales but are at different stages and Northern Ireland only began rolling out the programme in December 2009. Colonoscopy is offered to those who receive an abnormal FOB result. In the future, it is planned that one-off flexible sigmoidoscopy will be offered to everybody aged 55.

For bowel cancer screening we began follow-up for people at the latest date of registration at a general practice, their 60^th^ birthday or January 1^st^ 1998. Follow-up ceased at the earliest date of leaving the general practice, their 70^th^ birthday, of a medical record of colorectal cancer diagnosis or death.

#### 4) Prostate Screening Cohorts

The prostatic specific antigen (PSA) test is not currently recommended as a routine screening test for prostate cancer in the UK. However, the PSA informed choice programme involves GPs providing information to men who inquire about PSA testing and undertaking PSA tests with informed decision-making.

For the prostate screening cohort we began follow up for men at the latest date of their 50^th^ birthday, registering at a general practice, or January 1^st^ 1998. We ended follow-up at the earliest of leaving the general practice, their 70^th^ birthday, or when there was a GP record of prostate cancer diagnosis or death.

### Outcomes

The main outcomes for the study were a clinical record of 1) attending for cervical screening 2) attending for mammography (or a mammography result). 3) a Faecal Occult Blood Test result or 4) a PSA result.

### Covariates

We considered age, gender, and Townsend index as potential confounders. The Townsend index is a validated marker of the social deprivation of a geographical area [10]. In THIN, each patient is assigned a score according to their residential postcode in the UK. The data are available at a level of approximately 150 households and the patient is assigned a quintile of deprivation according to the deprivation of their area with national normative data as the reference group. The higher the deprivation quintile, the greater the social deprivation. Townsend scores are comprised of four indices of deprivation for an area; namely the percentage unemployed; the percentage of households who are homeowners and own a car, and level of overcrowding [10].

### Analysis

First we explored temporal trends in the recording of learning disability and the frequency with which different learning disability codes were used over time. We calculated incidence rates and rate ratios (IRRs) for receiving a screening test for each cancer using Poisson Regression, comparing the cohorts with and without learning disabilities. We produced unadjusted IRRs and then we adjusted the models for age, sex (only for bowel cancer since the other screens only apply to one sex) and country, and then for age, (sex), country and deprivation score. All models included the general practice as a random effect to account for clustering within practice. For the established screening programmes (cervical and breast), likelihood ratio tests were used to compare models with and without interactions between learning disability and 1) calendar period 2) age 3) deprivation quintile and 4) country. Where significant interactions were identified, stratified regression analyses were performed. Any time trends were explored using segmented regression analysis.

All analyses were performed using Stata version 12.

## Results

**Figure 1:**
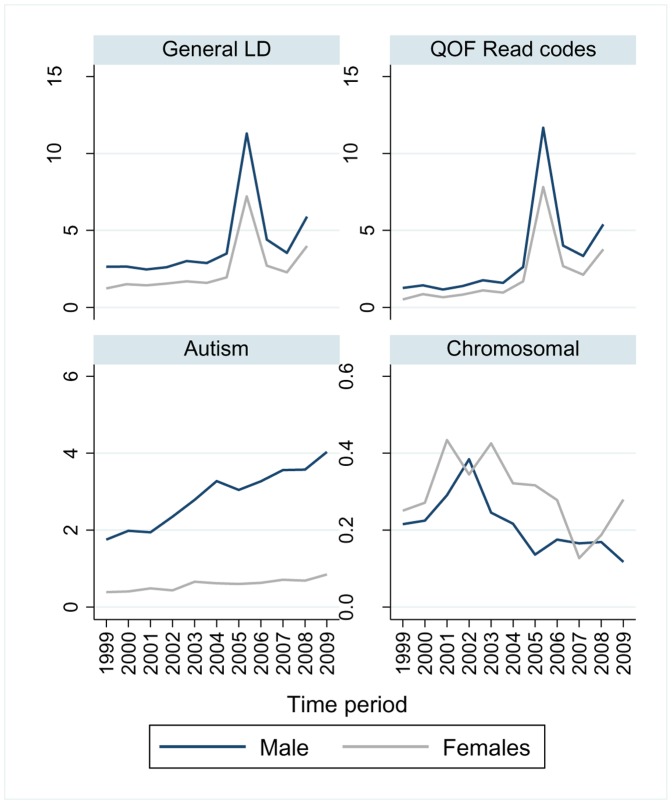
Time trends in use of diagnostic codes for learning disability in the THIN database 1999–2009. The figure charts time trends in the rates of recording of different diagnostic codes for learning disability (LD) by general practitioners in the UK THIN databse between 1999–2009. Separate graphs are provided for general learning disability diagnoses, autism and related diagnoses, chromosomal conditions associated with learning disability, and read codes used for the Quality Outcomes Framework (QoF). QoF is a financial incentive scheme to improve quality of primary care in England, and a register of those with learning disabilty became part of this scheme in 2006/7.

Time trends in the recording of learning disability within the THIN database are depicted in figure 1, spanning the study period between 1999 and 2009. Predictably there was a peak in recording Quality Outcomes Framework (QoF) codes for learning disability after the implementation of the learning disability QOF in 2006. There was a steady increase in the recording of autism across the decade which was more pronounced for males. Time trends in the use of codes for chromosomal syndromes related to learning disability showed no clear pattern and the observed changes may reflect general variance due to the smaller numbers of people involved.

Table 1 describes the cohorts with and without learning disability who were included in each of the cervical, breast, bowel and prostate screening comparisons. This includes their distribution by age, sex, deprivation score, year of entry to the cohort and country of residence in the UK. The total numbers of eligible people in the learning disability cohorts exceeded 6000 for the cervical and bowel screening comparisons with fewer people in the breast (n = 2956) and prostate (n = 3520) screening cohorts (Table 1).

**Table 1:** Description of cohorts with and without learning disability eligible to be screened for the four types of cancer.

	Cervical cohort		Mammogram cohort		PSA cohort		FOB cohort	
	No LD	LD	No LD	LD	No LD	LD	No LD	LD
Total Number	33,425	6,254	17,354	2,956	20,091	3,520	40,225	6,566
Mean age cohort entry (SD)	35.8 (10.3)	36.6 (11.1)	57.7(10.0)	57.5(10.0)	54.3 (5.1)	54.9 (5.7)	55.0 (5.8)	55.0 (5.7)
Median years follow-up time (IQR)	5.8 (2.6–9.7)	6.7 (3.2–10.4)	5.4 (2.4–9.4)	6.4 (2.9–10.3)	5.5 (2.5–9.4)	6.4 (3.0–9.9)	5.5 (2.4–9.4)	6.4 (3.0–10.1)
Males (%)	NA	NA	NA	NA	20,091	3,520	21810 (54)	3593 (55)
Females (%)	33,425	6,254	17,354	2,956	NA	NA	18415 (46)	2973 (45)
Time period (%)								
1999	6738 (20)	1472 (24)	3866 (22)	503 (17)	4758 (24)	653 (19)	9747 (24)	1223 (19)
2000	4090 (12)	708 (11)	1677 (10)	262 (9)	1926 (10)	321 (9)	3645 (9)	567 (9)
2001	3871 (12)	638 (10)	1637 (9)	232 (8)	1677 (8)	250 (7)	3485 (9)	465 (7)
2002	3519 (11)	624 (10)	1490 (9)	245 (8)	1667 (8)	262 (7)	3222 (8)	500 (8)
2003	3031 (9)	529 (8)	1639 (9)	275 (9)	1833 (9)	352 (10)	3641 (9)	627 (10)
2004	2641 (8)	483 (8)	1387 (8)	262 (9)	1630 (8)	315 (9)	3191 (8)	575 (9)
2005	2244 (7)	427 (7)	1336 (8)	251 (8)	1433 (7)	279 (8)	2893 (7)	524 (8)
2006	1915 (6)	298 (5)	1092 (6)	212 (7)	1277 (6)	250 (7)	2535 (6)	469 (7)
2007	2134 (6)	353 (6)	1162 (7)	239 (8)	1360 (7)	286 (8)	2738 (7)	540 (8)
2008	1831 (5)	362 (6)	1088 (6)	262 (9)	1307 (7)	297 (8)	2581 (6)	582 (9)
2009	1411 (4)	360 (6)	980 (6)	213 (7)	1223 (6)	255 (7)	2547 (6)	494 (8)
Social deprivation score (%)								
Missing	1464 (4)	253 (4)	624 (4)	127 (4)	720 (4)	165 (5)	1312 (3)	307 (5)
1 (least deprived)	7092 (21)	1027 (16)	4146 (24)	482 (16)	4757 (24)	455 (13)	9617 (24)	915 (14)
2	6554 (20)	991 (16)	3627 (21)	441 (15)	4384 (22)	639 (18)	8888 (22)	1081 (16)
3	6924 (21)	1246 (20)	3442 (20)	619 (21)	4002 (20)	687 (20)	8056 (20)	1334 (20)
4	6537 (20)	1437 (23)	3232 (19)	691 (23)	3582 (18)	843 (24)	7202 (18)	1561 (24)
5 (most deprived)	4854 (15)	1300 (21)	2283 (13)	596 (20)	2646 (13)	731 (21)	5150 (13)	1368 (21)
Country (%)								
England	25550 (76)	4789 (77)	13409 (77)	2285 (77)	15613 (78)	2717 (77)	31496 (78)	5102 (78)
Ireland	1661 (5)	292 (5)	732 (4)	129 (4)	809 (4)	147 (4)	1690 (4)	280 (4)
Scotland	4239 (13)	774 (12)	2182 (13)	370 (13)	2574 (13)	461 (13)	4795 (12)	812 (12)
Wales	1975 (6)	399 (6)	1031 (6)	172 (6)	1095 (5)	195 (6)	2244 (6)	372 (6)

LD Learning Disability PSA Prostate Specific Antigen Test FOB Faecal Occult Blood Test.

**Figure 2:**
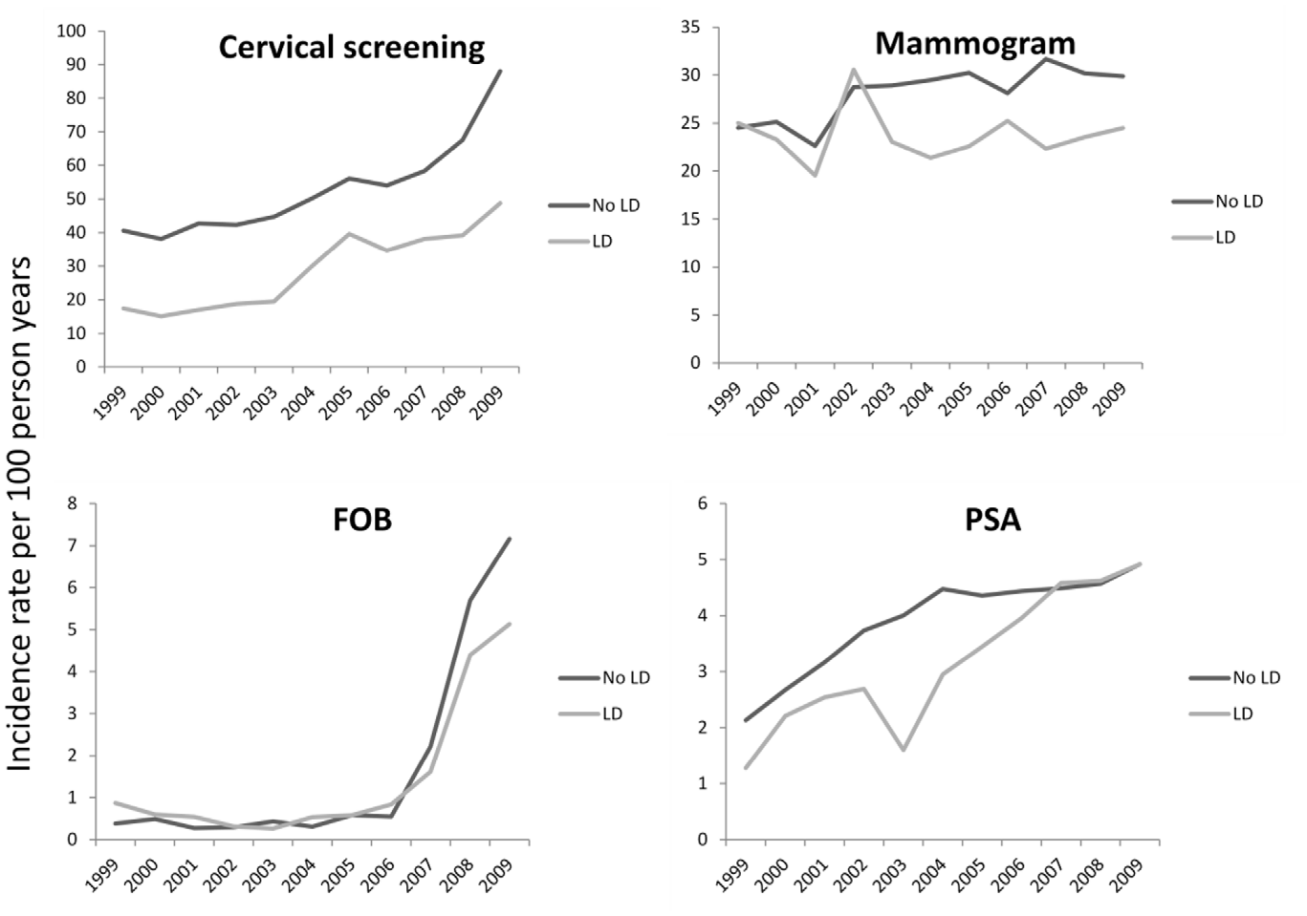
Incidence rates for screening for four types of cancer in people with and without Learning Disability. The figure compares rates of screening for cancer in eligible people with and without learning disability (LD), within the UK THIN primary care database from 1999–2009. The four charts relate to cervical screening rates, mammography rates, screening for bowel cancer with faecal occult blood (FOB) tests, and screening for prostate cancer with prostatic specific antigen tests (PSA).

The unadjusted incidence rates for cancer screening are presented in table 2 and figure 2. These screening *rates* are more informative than the absolute *numbers* screened since they account for the length of follow-up time available in THIN for each individual. The screening rates are presented by time period, Townsend quintile for deprivation and by country. Absolute rates of screening for all four cancers were lower in people with learning disability. Rates of recording of screening in THIN were comparable to rates published elsewhere. For those with at least 3 years follow-up, breast screening rates were 81% in the comparison cohort (compared with 75% in reported rates for the general population [14). Cervical screening rates in the comparison cohort were 97%.

**Table 2:** Incidence rates per 100 person years for cancer screening by time period, Townsend quintile for deprivation and by country.

	Cervical cohort		Mammogram cohort		PSA cohort		FOB cohort	
	No LD	LD	No LD	LD	No LD	LD	No LD	LD
Time period								
1999	40.65 (38.80–42.57)	17.44 (15.12–20.03)	24.53 (22.20–27.03)	25.02 (18.74–32.73)	2.13 (1.71–2.63)	1.28 (0.51–2.63)	0.38 (0.26–0.54)	0.87 (0.40–1.65)
2000	38.17 (36.68–39.70)	15.11 (13.27–17.13)	25.16 (23.20–27.24)	23.30 (18.45–29.04)	2.67 (2.27–3.14)	2.21 (1.31–3.49)	0.49 (0.37–0.64)	0.60 (0.27–1.14)
2001	42.72 (41.21–44.28)	17.02 (15.19–19.01)	22.64 (20.93–24.46)	19.53 (15.49–24.31)	3.17 (2.76–3.62)	2.54 (1.64–3.75)	0.28 (0.20–0.38)	0.54 (0.26–1.00)
2002	42.35 (40.89–43.85)	18.79 (16.97–20.76)	28.75 (26.90–30.68)	30.55 (25.72–36.03)	3.73 (3.32–4.18)	2.69 (1.83–3.82)	0.29 (0.21–0.39)	0.32 (0.13–0.65)
2003	44.70 (43.09–46.34)	19.51 (17.58–21.60)	28.91 (27.12–30.78)	23.05 (19.15–27.53)	4.01 (3.60–4.44)	1.60 (1.00–2.43)	0.44 (0.35–0.54)	0.27 (0.11–0.55)
2004	50.14 (48.37–51.96)	29.92 (27.46–32.54)	29.48 (27.67–31.37)	21.38 (17.75–25.52)	4.48 (4.06–4.92)	2.95 (2.16–3.93)	0.31 (0.24–0.40)	0.54 (0.31–0.87)
2005	56.07 (54.06–58.13)	39.64 (36.52–42.96)	30.22 (28.38–32.15)	22.59 (18.98–26.69)	4.35 (3.96–4.78)	3.45 (2.62–4.45)	0.58 (0.48–0.69)	0.58 (0.35–0.90)
2006	54.03 (51.87–56.25)	34.66 (31.44–38.12)	28.13 (26.30–30.05)	25.25 (21.37–29.63)	4.44 (4.04–4.86)	3.96 (3.08–5.01)	0.55 (0.46–0.65)	0.84 (0.56–1.20)
2007	58.36 (55.99–60.80)	38.12 (34.46–42.06)	31.67 (29.66–33.77)	22.34 (18.69–26.49)	4.49 (4.09–.91)	4.58 (3.64–5.70)	2.21 (2.03–2.41)	1.61 (1.22–2.08)
2008	67.64 (64.92–70.45)	39.18 (35.23–43.46)	30.17 (28.19–32.26)	23.56 (19.88–27.72)	4.57 (4.17–4.99)	4.62 (3.68–5.72)	5.70 (5.40–6.01)	4.40 (3.75–5.12)
2009	88.06 (84.39–91.84)	48.78 (44.09–53.82)	29.88 (27.85–32.01)	24.49 (20.69–28.79)	4.92 (4.50–5.36)	4.92 (3.95–6.05)	7.16 (6.82–7.52)	5.14 (4.43–5.92)
Social deprivation score								
1 (least deprived)	50.30 (49.06–51.55)	23.45 (21.68–25.32)	30.91 (29.67–32.19)	25.23 (21.88–28.95)	5.06 (4.77–5.36)	3.67 (2.85–4.65)	2.19 (2.06–2.32)	1.76 (1.37–2.23)
2	49.94 (48.66–51.25)	23.01 (21.24–24.88)	28.45 (27.21–29.74)	21.68 (18.53–25.21)	4.44 (4.17–4.74)	3.76 (3.06–4.56)	2.03 (1.90–2.16)	1.91 (1.54–2.35)
3	50.58 (49.30–51.89)	25.48 (23.77–27.29)	30.10 (28.73–31.53)	25.50 (22.64–28.62)	3.88 (3.61–4.17)	3.95 (3.26–4.75)	1.93 (1.80–2.07)	1.47 (1.18–1.80)
4	48.68 (47.39–49.99)	28.97 (27.22–30.80)	26.97 (25.66–28.32)	22.94 (20.49–25.61)	3.50 (3.22–3.80)	3.31 (2.75–3.94)	1.90 (1.76–2.05)	1.61 (1.33–1.92)
5 (most deprived)	46.17 (44.73–47.65)	30.59 (28.66–32.60)	23.23 (21.83–24.71)	23.38 (20.67–26.34)	3.02 (2.72–3.34)	2.98 (2.43–3.63)	1.76 (1.60–1.94)	2.00 (1.67–2.38)
Country								
England	49.84 (49.18–50.50)	27.35 (26.43–28.30)	31.91 (31.18–32.65)	27.17 (25.57–28.84)	4.20 (4.06–4.35)	3.44 (3.11–3.79)	2.32 (2.25–2.40)	1.91 (1.73–2.09)
Ireland	40.27 (38.15–42.47)	24.02 (20.70–27.72)	26.70 (24.30–29.27)	19.12 (14.75–24.37)	6.20 (5.42–7.05)	4.77 (3.22–6.81)	1.39 (1.15–1.65)	1.89 (1.23–2.77)
Scotland	53.35 (51.62–55.13)	27.68 (25.32–30.20)	16.94 (15.84–18.10)	12.96 (10.70–15.55)	2.51 (2.24–2.81)	2.66 (2.01–3.45)	0.46 (0.38–0.56)	0.95 (0.66–1.32)
Wales	47.70 (45.38–50.10)	18.89 (16.41–21.63)	19.72 (17.85–21.74)	14.68 (10.82–19.46)	5.14 (4.53–5.81)	5.17 (3.69–7.04)	0.97 (0.79–1.17)	0.81 (0.43–1.38)

LD Learning Disability, PSA Prostate Specific Antigen Test, FOB Faecal Occult Blood Test. (95% confidence intervals).

The results of the poisson regression analysis (using the whole sample) are presented in table 3, with incidence rate ratios for cancer screening. The analysis confirmed that people with learning disability were significantly less likely to receive screening for all four cancers we studied. Accounting for age, period and country had minimal impact on the results.

**Table 3:** Incidence rate ratios for receiving screening for cervical, breast, bowel and prostate cancer in people with and without learning disability. Results from Poisson regression.

								P-values for interaction terms with LD			
		No. event	Person years	IR per 100 PYs(95%CI)	Unadjusted IRR(95%CI)	Adjusted IRR (95%CI):age, period, country, sex*	Adjusted IRR (95%CI): age, period, country, sex*, Townsend	Period	Age	Townsend	Country
**Cervical smear**	**No LD**	28512	57500	49.6 (49.0–50.1)	0.55 (0.53–0.57) p<0.001	0.54 (0.52–0.56) p<0.001	0.54 (0.52–0.56) p<0.001	p<0.001	NS	p<0.001	p<0.001
	**LD**	4236	15900	26.6 (25.8–27.5)							
**Mammogram**	**No LD**	9000	31708	28.4 (27.8–29.0)	0.78 (0.74–0.83) p<0.001	0.75 (0.71–0.80) p<0.001	0.76 (0.72–0.81) p<0.001	0.002	NS	0.008	NS
	**LD**	1309	5533	23.7 (22.4–25.0)							
**PSA**	**No LD**	3931	95552	4.11 (3.99–4.24)	0.84 (0.77–0.92)p<0.001	0.83 (0.76–0.91) p<0.001	0.87 (0.8–0.96) p = 0.004				
	**LD**	530	15255	3.47 (3.19–3.78)							
**FOB**	**No LD**	4119	20586	2.00 (1.94–2.06)	0.87 (0.80–0.96)p = 0.004	0.84 (0.77–0.92) p<0.001	0.86 (0.78–0.94) p = 0.001				
	**LD**	518	2995	1.73 (1.59–1.88)							

* FOB adjusted for gender.

NS = non statistically significant (p>0.05).

LD Learning Disability, PSA Prostate Specific Antigen Test, FOB Faecal Occult Blood Test, CI Confidence Interval, IR Incidence Rate Ratio, IRR Incidence Rate Ratio.

Exemption from cervical cancer screening was more common in people with learning disability, 25% compared to 6% in the comparison cohort. Where recorded, the most common reasons were refusal or being deemed inappropriate for screening. Our main analysis omitted these excluded patients. Before accounting for this exclusion reporting, the people with learning disabilities were 56% less likely to be screened than their counterparts of normal intelligence (unadjusted IRR for cervical screening: 0.44, 95%CI; 0.42–0.46). However, recorded exclusions did not fully account for the difference in cervical screening rates. In our main analysis, people with learning disability were 45% less likely to be screened (Table 3: unadjusted IRR for cervical screening = : 0.55, 95%CI; 0.53–0.57).

The analysis of interactions for the established screening programmes identified significant differences according to time period, Townsend score and for country (cervical screening only). There was less disparity in cancer screening among people living in the most deprived areas of the UK (tables 2 and 3); in fact discrepancies in screening rates were more pronounced in the least deprived areas of each country. Cervical and breast screening were relatively less common in people with learning disability in all four countries of the UK (Tables 2 and 3). These interactions were most consistent for cervical cancer screening and stratified analyses are presented in table 4. Differences in rates of cervical cancer screening became less pronounced over the decade, and the time trend analysis derived three distinct time trend periods (shown in table 4). In 2008/9 people with learning disability were still 45% less likely to be screened that their counterparts without learning disability (Table 4: adjusted IRR for cervical screening in 2008/9∶0.55 (0.53–0.57)). Although the differences in mammography screening showed less consistent trends statistically, they appeared to widen over the decade, (IRR in 1999∶0.84; 0.62–1.13. IRR in 2009∶0.65; 0.54–0.78). Disparities in screening rates for both cancers were least pronounced in the more socially deprived areas (cervical cancer; table 4). For breast screening the IRR in the least deprived areas was 0.69 (0.59–0.80), in contrast to 0.93 (0.80–1.08) in the most deprived areas. The differences in cervical screening rates for people with and without learning disabilities were similar in England, Ireland and Scotland but the difference appeared to be slightly greater in Wales (Table 4).

**Table 4:** Adjusted incidence rate ratios for receiving screening for cervical cancer with and without learning disability stratified on variables where significant interactions with learning disability status were identified (see Table 3).

	LD vs. non LD		
	Adjusted* IRR	95%CI	p-value
Time period			
1999–2004	0.45	0.43–0.47	<0.001
2005–2007	0.60	0.56–0.65	<0.001
2008–2009	0.55	0.51–0.61	<0.001
Townsend score			
1	0.45	0.42–0.49	<0.001
2	0.45	0.41–0.49	<0.001
3	0.51	0.48–0.55	<0.001
4	0.60	0.56–0.65	<0.001
5	0.65	0.60–0.70	<0.001
Country			
England	0.55	0.53–0.57	<0.001
Ireland	0.57	0.49–0.67	<0.001
Scotland	0.53	0.48–0.58	<0.001
Wales	0.43	0.37–0.49	<0.001

* Adjusted for all other variables except the stratifying variable (age, time period, Townsend score and country).

IRR Incidence rate ratio.

## Discussion

People with a diagnosis of learning disability in primary care have fewer recorded screening tests for both cervical and breast cancer, compared to demographically similar general practice comparison patients. They are also less likely to have a record of testing for faecal occult bloods or prostatic specific antigen. Over the decade we studied, differences in rates of cervical cancer screening narrowed, but by 2008/9 people with learning disability were still 45% less likely to receive a screen. Rates of mammography recording showed less consistent patterns, in 2009 people with learning disability were 35% less likely to receive a mammogram than the comparison group. Differences in rates of screening for breast and cervical cancer are significantly more prominent for people living in less socially deprived geographical areas. For cervical cancer, people with learning disability are less likely to be screened than the general population if they live in more affluent areas. For breast cancer, the rates of screening for people with learning disability show less variation by social deprivation. Our findings differed slightly in the four countries of the UK. In particular relative rates of cervical cancer screening were significantly lower for people with learning disability in Wales.

### Strengths and Limitations

Our study included a large representative sample of people with learning disability in the UK and we employed robust methodology to define the cohorts with and without learning disability who are eligible for screening for four different types of cancer, in accordance with UK health policy. The cohort design accounted for the amount of follow-up time available for each patient within the database, and we restricted our analysis to four main outcomes and a limited number of tests for interactions which were pre-determined by clinical hypotheses, namely that differential rates would vary over time, by area social deprivation and by country. Our study only includes people who have received a general practice code for learning disability and it is possible that some people with such a disability were not identified. Severity of learning disability was infrequently recorded by GPs and therefore we were not able to explore the influence of the severity of the condition on rates of cancer screening. The bowel and prostate cancer screening schemes are relatively recent initiatives in the UK and it maybe that further disparities may emerge as they become more established with the UK population.

### Clinical Implications

People with learning disability are less likely to have a record of screening for breast, cervical, prostate and bowel cancer than general practice patients of similar age and sex without learning disabilities. The cervical screening results concur with a recent NHS report regarding this group of patients [15]. However we have provided stronger evidence by using more contemporary data and by adjusting for follow-up time in THIN, by exploring time trends and also evaluating breast, bowel and prostate cancer screening. Interestingly, in the NHS report, rates of screening for other conditions such as obesity, hypertension and diabetes were similar in people with and without learning disability [15]. Therefore it appears there may be a specific challenge relating to cancer screening in this group.

Explanations may lie at the level of the individual patient, their carers, their health professionals, health policy or indeed the uptake of screening by those in the comparison cohorts. When we explored rates of exclusion from cancer screening, it was apparent that primary care physicians are more likely to exclude people with learning disability from being eligible for cervical cancer screening. However we accounted for this in our analysis, and people with learning disability were still less likely to be screened. It is also possible that patients do not receive appropriate information that encourages them to be screened for cancer, or that they choose not to be screened even if they do receive such information. In our study, the UK Department of Health guidance to improve cervical and breast screening in people with learning disability did not have a consistent discernible impact, since while relative rates of cervical screening improved, the breast screening gap has widened over the decade. Differences in the timing and nature of screening programmes in the four countries of the UK might explain the different findings we observed in the four countries of the UK. The NHS is committed to addressing health inequalities and the differential rates of cancer screening in people with learning disability need greater publicity and ongoing monitoring to avoid preventable physical morbidity. Other methods of reducing inequalities in access to cancer screening for this group should be considered.

The findings regarding social deprivation findings were contrary to our hypotheses. They may reflect a combination of a relatively higher uptake of screening by more affluent people without learning disability and in contrast a relatively lower uptake of screening in more deprived areas by people without learning disability. An alternative explanation is that people with learning disability may receive better care within geographical areas which are more socially deprived. We have observed similar findings when exploring cardiovascular screening for people with severe mental illnesses such as schizophrenia [16]. Contrary to intuition, differential screening rates were not more pronounced in areas of greater social deprivation, and it is possible that services in more deprived areas are more acclimatised to providing care for hard-to-reach groups.

We have detected discrepancies in the rates of cancer screening for people with learning disability, but analysis of routinely collected clinical data cannot fully determine the reasons for these inequalities. This would require primary research exploring the reasons why uptake of screening differs in these groups. One possibility is that people with learning disability have more physical co-morbidities which might make it more difficult for them to take up screening, but we did not have data to explore this possibility.

Furthermore, our research did not focus on a physical health outcome (ie cancer) within the cohorts and future research should determine whether the differential rates of screening are reflected by increased incidence rates for cancer in people with learning disability.

Our research focussed on the NHS cancer screening programmes and it is worth noting that while the effectiveness of cervical screening is well established for women over the age of 25 [17], there are still debates regarding the risks and benefits of breast cancer screening programmes [18], and even more uncertainty regarding the benefits of PSA tests in terms of prostate cancer screening [19]. However we sought to determine whether people with learning disability were receiving the screening recommended for people of their agegroup and they were not. As evidence emerges regarding the risks and harms of different cancer screening tests, the nature of the cancer screening programmes will inevitably change. However as a general principle, people with learning disability should have similar access to the best current physical health care as the general population. Although practitioners might be concerned regarding the possible psychological harm of cancer screening for vulnerable groups such as those with learning disability, this is not in line with national guidance for screening for this group of people [8].

Our results suggest that the introduction of various primary care incentives such as the NHS Quality Outcomes Framework and directed and local enhanced service schemes over the years may have narrowed the differences in screening for cervical cancers in people with learning disabilities but this is not the case for breast cancer screening. Moreover, for all four type of cancer screening there was a wide gap in the level of provision. Increased awareness of general practice staff to these discrepancies is required and the inclusion of more focussed cancer screening programmes as a part of the either the NHS Quality Outcomes Framework or the NHS Directed Enhanced Service schemes should be considered.
